# Nutrient intake, digestibility, and utilization in goats fed graded levels of hempseed cake finisher diets

**DOI:** 10.1007/s11250-023-03864-1

**Published:** 2023-12-19

**Authors:** Farouk Semwogerere, Obert C. Chikwanha, Chenaimoyo L. F. Katiyatiya, Munyaradzi C. Marufu, Cletos Mapiye

**Affiliations:** 1 Department of Animal Sciences, Faculty of AgriSciences, Stellenbosch University, Private Bag X1, Matieland, 7602 South Africa; 2 Department of Veterinary Tropical Diseases, Faculty of Veterinary Science, University of Pretoria, Onderstepoort, 0110 South Africa

**Keywords:** Hempseed cake, Nutrient digestibility, Purine derivatives, Volatile fatty acids

## Abstract

Globally, the price of soybean meal, the most common proteinaceous ingredient in livestock diets, has become highly expensive prompting a search for alternative ingredients. Hemp seed cake is a promising alternative but could be limited by its high neutral detergent fiber and ether extract contents which impede nutrient intake and digestibility. However, some ruminant species such as goats have superior ability to digest high fiber and ether extract diets. Thus, the current research evaluated nutrient intake and digestibility, rumen fermentation, and microbial protein synthesis of goats fed hempseed cake as a substitute for soybean meal in finisher diets. A total of 25 Kalahari Red castrates (27 ± 3 kg, 4–5 months old) were assigned to five dietary treatments (5 goats/ diet) in a completely randomized design. A maize-lucerne-based finishing diet was formulated with hempseed cake substituting soybean meal as the primary protein ingredient at 0, 25, 50, 75, or 100 g/kg dry matter. Ether extract intake exhibited a positive linear trend (*P* ≤ 0.05) while crude protein intake and microbial nitrogen supply exhibited a negative linear trend (*P* ≤ 0.05) with dietary inclusion of hempseed cake. However, feeding hempseed cake did not influence (*P* > 0.05) apparent nutrient digestibility, rumen fermentation parameters and nitrogen use efficiency. In conclusion, the substitution of soybean meal for hempseed cake decreased crude protein intake and microbial nitrogen supply in goat finisher diets without compromising nutrient digestibility and nitrogen use efficiency. The study recommends partial or full replacement of soybean meal with hempseed cake in goat finisher diets.

## Introduction

Globally, the production of hemp (Cannabis sativa L.) is increasing due to the legalization of its cultivation and revived fiber, food and medicinal uses (Siano et al. 2019; Horne 2020; Leonard et al. 2020). About 143 metric tons (MT) of hempseed are produced annually yielding about 50 MT of oil and 93 MT of cake worldwide (FAOSTAT 2022). Hempseed by-products (i.e., cake and hulls) have potential economic uses, including feed, human food and beverages (Frassinetti et al. 2018; Leonard et al. 2020; Aloo et al. 2022). Valorization of hempseed by-products as animal feedstuff provides a potential alternative to increasing their economic value, thus improving the importance of hempseed by-products in the circular bioeconomy (Moscariello et al. 2021; Aloo et al. 2022; Winders et al. 2023).

Hempseed cake (HSC) has the highest potential among the hemp by-products to be used as animal feedstuff owing to its high crude protein (CP; 341 ± 50.4 g/kg dry matter; DM), neutral detergent fiber (NDF; 395 ± 40.7 g/kg DM) and ether extract (EE; 116 ± 15.5 g/kg DM) with the NDF and EE almost triple that of soybean meal (SBM; NDF: 125 ± 17.6; EE: 40 ± 15.9; g/kg DM) (Pojić et al. 2014; Paula et al. 2018; Leonard et al. 2020; Semwogerere et al. 2023a). Apart from having high protein and energy contents, HSC contains bioactive compounds such as cannabinoids, tocopherols and polyunsaturated fatty acids (PUFA) (Leonard et al. 2020; Sainz Martinez et al. 2023; Semwogerere et al. 2023b).

Previous reports in ruminants indicate that high NDF (> 355 g/kg DM) and EE (> 50 g/kg DM) rich in PUFA inhibit nutrient intake, digestion and utilization among ruminants (Pantoja et al. 1994; Harper and McNeill 2015; Wang et al. 2017; Embaby et al. 2019). However, goats might be able to digest HSC diets better than other ruminants as they have better feed grinding ability and spend more time chewing and ruminating, which gives them the capacity to digest fibrous diets (Domingue et al. 1991; Lu et al. 2008). More so, goats tend to have more *Butyrivibrio fibrisolvens* that enhance fibre digestion in the rumen than the other ruminants (Candyrine et al. 2017).

Soybean meal, the primary protein source in goat finisher diets is normally included at 120 g/kg DM (Brand et al. 2020; Kustantinah et al. 2020; Dahmer et al. 2022). Replacing SBM with HSC (100 g/kg DM) linearly decreased in vitro DM digestibility but maintained the DM intake of goats (Abrahamsen et al. 2021; Semwogerere et al. 2022). More importantly, there is an information gap on the level of HSC in goat diets beyond which nutrient intake, digestibility and utilization efficiency are compromised. It was hypothesized that replacing SBM with increasing proportions of HSC up to 100 g/kg DM in goat finishing diets could maintain nutrient supply, digestion, and utilization. Thus, the current study investigated nutrient intake and digestibility, ruminal fermentation parameters, and microbial protein synthesis in goats fed pelleted finisher diets containing HSC substituting SBM.

## Materials and methods

### Research site

Welgevallen Experimental Farm, Stellenbosch University, Stellenbosch, South Africa, hosted the research study during June and July 2021. The experimental site experiences a Mediterranean climate and was characterized by an average temperature of 12°C, rainfall of 141 mm, and humidity of 77% during the experimental period.

### Goat diets and management

A local oilseed processing company (SeedOil^SA^, Somerset West, Cape Town) supplied cold-pressed HSC (*Cannabis Sativa* L. Fedora 17) which was used to formulated five maize-lucerne based goat finisher diets to satisfy the dietary needs for growing goats (CP: 120 g/kg DM and ME: 11.0 MJ/kg DM; National Research Council 2007). The milled (1.5-mm sieve) HSC was included at levels of 0, 25, 50, 75 and 100 g/kg DM replacing SBM and pelleted (5 mm × 30 mm) at 45°C (Table 1). The hay and straw were milled through 4 mm sieve prior to mixing. A local goat producer supplied clinically healthy 4–5 months Kalahari Red wether goats (27 ± 3 kg). Prior to purchase, the goats were raised on natural pasture and offered protein supplements in summer. In the study, goats were reared and fed individually (1 m × 2 m) with visual contact on a wooden slatted floor pen in a barn. Each dietary treatment received five goats in a completely randomized design thus 25 goats were used. Upon arrival, a 2 mL Multivax P-Plus (MSD, South Africa) was used to vaccinate goats against pasteurellosis, pulpy kidney and tetanus. Thereafter, a multivitamin (Embavit™®, Prima Vetcare Private Limited, India) dose of 5 mL was administered. Goats were drenched with 2 mg/kg bodyweight derquantel and 0.2 mg/kg bodyweight abamectin (Startect®, Zoetis, South Africa) for internal parasite control. Goats were adapted to the experimental diets for 21 days, data was collected for 5 days, and then slaughtered at an abattoir in accordance with the South African Meat Safety Act (No. 40 of 2000). A daily feed of 110% of the previous day's intake was offered to goats, and clean fresh water was available ad libitum.

**Table 1:** Feed ingredient proportions in experimental diets

Ingredients (g/kg DM)	Hempseed cake inclusion level (g/kg DM) in the diet				
	0	25	50	75	100
Soybean meal	100	75	50	25	0
Hempseed cake	0	25	50	75	100
Maize white fine	400	400	400	400	400
Lucerne hay	200	200	200	200	200
Hominy chop	93.5	93.5	93.5	93.5	93.5
Molasses syrup	70	70	70	70	70
Wheat straw	50	50	50	50	50
Wheat bran	49	49	49	49	49
Soybean hulls	10.6	10.6	10.6	10.6	10.6
Savannah Lime	7	7	7	7	7
Vitamin-mineral premix*	7	7	7	7	7
Ammonium sulfate	5	5	5	5	5
Coarse salt	4.9	4.9	4.9	4.9	4.9
Urea	3	3	3	3	3

*The composition of the vitamin/premix was not included because of a non-disclosure agreement with the feed manufacturer

Proportions of the feed ingredients in the experimental diets presented in this Table 1 are the same with those reported by Semwogerere et al. (2022, 2023a)

### Measurements and sample collection

Fecal collection bags were strapped on and funnel-shaped latex bags were attached to urinary tubes on each goat. Before daily feeding, 10% of the feces and spot urine were collected and stored at -20°C pending analysis. Feed, refusal and fecal samples collected per animal daily for 5 days were later composited into one sample per animal for the analyses. Feed, refusal and fecal samples were milled through a 1.5-mm sieve after being dried in a forced-air ventilation oven at 60°C for 72 h.

To protect the purine derivatives (PD) from bacterial destruction and ammonia from volatilization, spot urine was collected in a 4-L bottle containing 100 mL of 10% (v/v) sulfuric acid to maintain the pH below 3. A subsample of 20 mL was diluted 5-folds with distilled water for PD and 80 mL was undilutedly kept for nitrogen analysis. The urine samples were then stored at -20°C pending analyses. A composite of 5 days urine collection per animal was stored.

Exactly, 15 min postmortem, rumen contents were emptied into a bucket and blended prior to measuring pH (Crison PH25 pH meter, Lasec, South Africa). Four layers of muslin butter cloth and glass wool were used to filter the rumen contents into 50 mL Greiner centrifuge tubes (Merck KGaA, Darmstadt, Germany). The supernatant was obtained after centrifuging the rumen fluid at 1000 g for 10 min (4°C), then stored at -20°C.

### Chemical analyses and computations

#### Chemical composition and fiber

The feed, refusal and fecal DM, ash, EE, CP, starch, total phenols and tannins were analyzed according to methods 934.01, 942.05, and 920.39, respectively in the AOAC (2002) procedure. The total nitrogen content of feed, refusal, fecal and urine was determined using a macro-Nitrogen analyser (LECO® FP828, LECO Corporation, Miami, USA) with a dumas method of AOAC (2002) and a factor of 6.25 was used to obtain the CP content. The procedure described by Hall (2009) was used to estimate the total starch content of the samples with glucose and/ or maltodextrins using a commercial starch assay (Total Starch Megazyme kit KTSTA, Megazyme International Ireland Ltd., Wicklow, Ireland). Total phenols and tannins were measured using the Folin-Ciocalteu colorimetric method described by Makkar (2003). The F57 filter bags and Ankom fiber analyser 2000 (ANKOM Technology, NY, USA) were used to measure neutral detergent fiber (aNDFom), acid detergent fiber (ADFom) and lignin (sa.) calculated without ash (Ryan et al. 1990).

A multispecies Ncal™ International Standard Direct® urinary creatinine detection kit (K002-H1, Arbor Assays Ann Arbor, Michigan, USA) was used to determine the creatinine concentration colorimetrically (SPECTROstar Nano, BMG LABTECH, Germany). An average of 17.39 mg/kg bodyweight (BW) of urinary creatinine excretion by each goat was used (Santos et al. 2017). The total daily urine output was estimated using the formula: (BW × 17.39 × 100)/spot urine creatinine concentration mg/dL of urine (Costa et al. 2021). The formula used to determine apparent nutrient digestibility is [(nutrient intake (g/day) - nutrient in fecal matter (g/day)] × 100 (Wiseman 2018). Ammonia-nitrogen concentrations were determined using the colorimetry technique (Broderick and Kang 1980), and volatile fatty acids were measured using the Siegfried et al. (1984) methodology with gas chromatography.

### Urinary purine and microbial nitrogen

Using methods described by Chen and Gomes (1992), allantoin was determined colorimetrically, uric acid using uricase and xanthine plus hypoxanthine using xanthine oxidase. The sum of the amounts of allantoin, uric acid, xanthine, and hypoxanthine excreted in the urine was used to compute the total quantity of PD discharged. According to Chen and Gomes (1992), the quantitative association between intestinal flow of microbial nitrogen, PD excretion in urine, and microbial purine absorption was estimated. The difference between daily nitrogen intake and daily nitrogen excretion was used to compute nitrogen retention (g/d). By dividing nitrogen retained with nitrogen intake, efficiency of nitrogen utilization (%) was calculated.

### Validation and quality assurance

The number of replicates were selected in accordance with Ricci et al. (2020) for animals and Udén et al. (2012) for feed samples. The allocation of animals to experimental diet and housing pens as well as samples for analysis was randomized using RAND function of MS excel. The proximate and fiber analyses were executed in triplicate while rumen fermentation parameters and purine derivatives were run in duplicate. The proximate and fiber analyses were run with a known control sample while rumen fermentation parameters and purine derivatives were run with a standard. The analytes were considered valid when the value of the standard deviated by <3%.

### Statistical analyses

Normality and homoscedasticity of the data were checked using the Univariate procedure with the normal statement (version 9.4; SAS Institute Inc. Cary, NC, USA). All the data conformed to normality following the removal of outliers according to Shapiro and Wilk (1965). The GLIMMIX procedure (version 9.4; SAS Institute Inc. Cary, NC, USA) was used to analyze data. The model included diet as the main factor. Each goat was treated as an experimental unit. The standard model used was as follows: yijk = μ+ Ti + εijk, where yijk = represented the kth observation on the jth goat assigned to ith diet, μ = overall means; Ti = was the fixed effect of the ith diet (0, 25, 50, 75, and 100 g/kg DM HSC) and εijk = was the residual error at kth observation on jth goat and ith diet (0, 25, 50, 75, and 100 g/kg DM HSC). Polynomial contrasts were performed to determine the linear and quadratic trends of dietary inclusion levels. A Tukey’s test was used to declare least square means significantly different at P ≤ 0.05 and tendencies at 0.05 < P ≤ 0.10.

## Results

### Feed ingredients and diet chemical attributes

Diets, HSC, SBM, and ration formulations all have chemical profiles that are similar to those reported by Semwogerere et al. (2022, 2023a, 2023b) (Tables 1 and 2). In summary, the inclusion of HSC in diets resulted in a linear increase (P ≤ 0.05) in DM, EE and ash, fiber (i.e., NDF, ADF and lignin) and phenolic components (i.e., total phenols and tannins) and linear decrease (P ≤ 0.05) in CP, starch, metabolizable energy and non-fibrous carbohydrates.

**Table 2:** Chemical composition of dietary ingredients and experimental diets

Chemical Composition (g/kg DM unless stated)	Hempseed cake	Soybean meal	SEM^7^	Hempseed cake inclusion level (g/kg DM) in the diet					SEM^7^	*P*-value		
				0	25	50	75	100		Diet	Linear	Quadratic
Dry matter (DM)	926	899	3.52	877	882	882	887	887	1.14	0.001	0.001	0.238
Ash	155	83.7	5.76	53.9	55.3	55.8	57.8	58.7	0.73	0.001	0.001	0.802
Crude protein (CP)	246	369	3.17	132	133	130	130	124	0.89	0.002	0.001	0.052
Starch	76.5	91.5	4.69	406	346	321	251	222	13.71	0.001	0.001	0.744
Ether extract (EE)	80.6	6.87	0.27	25.0	27.1	28.8	29.7	33.1	0.44	0.001	0.001	0.294
Neutral detergent fiber (aNDFom)^1^	374	67.2	3.34	168	169	171	174	176	1.75	0.006	0.001	0.578
Acid detergent fiber (ADFom)^2^	255	51.8	2.88	90.1	91.0	97.9	98.8	102.4	2.13	0.001	0.001	0.952
Lignin (sa)^3^	91.3	10.3	1.48	16.2	16.2	16.9	19.1	19.2	0.32	0.001	0.001	0.212
Metabolizable energy (MJ/kg)^4^	10.2	11.4	0.10	12.0	12.0	11.9	11.8	11.78	0.03	0.001	0.001	0.637
Non-fibrous carbohydrates (NFC)^5^	145	473	6.05	621	616	614	608	607	2.04	0.001	0.001	0.554
Total phenols (g GAE/kg DM)^6^	0.36	0.16	0.02	0.4	0.4	0.40	0.4	0.4	0.01	0.001	0.020	0.857
Tannins (g GAE/kg DM)^6^	0.25	0.07	0.02	0.231	0.240	0.255	0.257	0.263	0.001	0.036	0.002	0.487

All chemical analyses were analyzed in triplicate with five replicates per sample

^1^aNDFom: Neutral detergent fiber analysed with a heat-stable amylase and reported without ash

^2^ADFom: Acid detergent fiber reported without ash

^3^Lignin (sa.): Lignin analysed by solubilization of cellulose with sulfuric acid

^4^Calculated according to Freer et al. (2007)

^5^Non-fibrous carbohydrates: calculated as: 1000 – (aNDFom + Crude protein + ether extract + ash; g/kg)

^6^GAE: Gallic acid equivalent

^7^SEM: Standard error of means

Chemical composition of dietary ingredients presented in this Table 2 is the same with those reported by Semwogerere et al. (2022, 2023b)

### Nutrient intake, digestibility, and ruminal fermentation parameters

Table 3 shows the effects of feeding graded HSC levels to Kalahari Red goats on nutrient intake and digestibility. The DMI (P = 0.081) and organic matter (OM) intake (P = 0.067) tended to decline linearly with dietary inclusion of HSC. Crude protein intake decreased linearly (P ≤ 0.05) while EE intake increased linearly (P ≤ 0.05) with dietary inclusion of HSC. Diet had no effect (P > 0.05) on apparent total digestibility parameters. The effects of feeding increasing levels of HSC to Kalahari Red goats on rumen fermentation parameters are shown in Table 4. The ruminal fermentation parameters were not affected by diet (P > 0.05), except for ruminal pH (P = 0.090) which tended to increase linearly with dietary inclusion of HSC.

**Table 3:** Effects of feeding increasing levels hempseed cake on nutrient intake and digestibility of wether goats

	Hempseed cake inclusion level (g/kg DM) in the diet						*P*-value		
	0	25	50	75	100	SEM	Diet	Linear	Quadratic
Nutrient intake (g/d)									
Dry matter	1080	1039	1012	1012	964	44.3	0.472	0.081	0.941
Organic matter	1013	973.4	947.9	946.3	899.9	41.5	0.431	0.067	0.944
Crude protein	162.7	156.3	149.3	148.9	135.1	6.61	0.081	0.007	0.737
Neutral detergent fiber (aNDFom)	206.7	199.2	196.3	199.1	192.2	8.54	0.815	0.293	0.831
Ether extract	30.7	31.9	33.1	33.9	36.0	1.38	0.121	0.011	0.769
Apparent total tract digestibility^a^									
Dry matter	73.1	73.1	73.1	74.1	74.1	2.00	0.987	0.625	0.893
Organic matter	75.5	74.9	74.8	75.5	74.7	1.87	0.996	0.882	0.965
Crude protein	72.0	71.9	71.8	74.1	74.8	2.16	0.789	0.269	0.628
Neutral detergent fiber (aNDFom)	58.3	58.0	55.9	55.7	55.3	2.48	0.893	0.365	0.941
Ether extract	83.6	84.0	84.0	84.7	84.8	1.40	0.971	0.497	0.972
True organic matter digestibility	91.5	91.4	90.9	90.9	90.5	0.52	0.614	0.130	0.945

^a^Apparent digestibility = [(nutrient intake (g/day) - nutrient in fecal matter (g/day))/ nutrient intake (g/day)] × 100

*SEM* standard error of means

**Table 4:** Effects of feeding increasing of hempseed cake on ruminal fermentation parameters of wether goats

	Hempseed cake inclusion level (g/kg DM) in the diet					SEM	*P*-value		
	0	25	50	75	100		Diet	Linear	Quadratic
pH	6.6	6.7	6.8	6.8	6.8	0.09	0.473	0.090	0.674
Ammonia-nitrogen (mg/L)	322	324	331	327	376	50.12	0.931	0.481	0.665
Total volatile fatty acids (VFA, mM)	56.5	57.6	59.2	63.5	66.8	7.21	0.836	0.260	0.797
VFA (mM/ 100 mM)									
Acetate	64.8	62.5	63.6	65.2	62.6	2.68	0.926	0.829	0.987
Propionate	18.3	19.2	19.0	18.6	21.1	1.69	0.793	0.354	0.640
Butyrate	12.1	13.3	12.1	11.2	10.7	1.76	0.870	0.409	0.658
Isobutyrate	1.6	1.7	1.5	1.4	1.3	0.24	0.822	0.292	0.673
Valerate	1.18	1.13	1.32	1.25	1.38	0.25	0.955	0.517	0.921
Isovalerate	2.07	2.16	2.46	2.35	2.84	0.49	0.824	0.281	0.830
Acetate: propionate ratio	3.66	3.39	3.36	3.81	3.13	0.46	0.851	0.672	0.843

*SEM* standard error of means

### Purine derivatives and nitrogen balance

Feeding HSC reduced (*P* ≤ 0.05) total urine output in a linear fashion (Table 5). The inclusion of HSC in goat finisher diets linearly decreased (P ≤ 0.05) urinary excretion of allantoin and xanthine plus hypoxanthine (Table 5). Microbial nitrogen supply, total PD absorbed and excreted, intake and urinary nitrogen decreased linearly (P ≤ 0.05) with increasing dietary inclusion levels of HSC (Table 5). Fecal N, retention N and utilization efficiency of N were not influenced (P > 0.05) by diet (Table 5).

**Table 5:** Effects of feeding increasing of hempseed cake on purine derivatives, microbial nitrogen supply and nitrogen retention of wether goats

Variables	Hempseed cake inclusion level (g/kg DM) in the diet					SEM	*P*-value		
	0	25	50	75	100		Diet	Linear	Quadratic
Urine (mL/d)	1062.0	936.5	673.6	658.3	639.1	144.79	0.173	0.023	0.406
Urinary excretion (mM/d)									
Allantoin	27.2	19.0	17.2	16.6	14.1	3.17	0.076	0.001	0.300
Uric acid	0.8	0.7	0.7	0.7	0.7	0.15	0.991	0.662	0.845
Xanthine + Hypoxanthine	3.0	2.1	1.6	1.0	0.8	0.37	0.002	0.001	0.436
Total purine derivatives excreted	22.3	20.8	18.5	18.1	18.6	1.43	0.200	0.036	0.269
Total purine derivatives absorbed	24.2	22.4	19.6	19.1	19.8	1.70	0.200	0.036	0.269
Microbial nitrogen supply, g N/day DOMI	16.6	15.3	13.3	12.9	12.8	1.23	0.148	0.018	0.387
Nitrogen balance (g/day)									
N intake	42.6	40.3	37.2	36.7	34.4	1.26	0.002	0.001	0.572
Fecal N excretion	16.7	15.1	16.2	17.4	15.4	1.81	0.897	0.977	0.934
Urinary N excretion	13.9	11.1	8.1	7.6	7.2	1.82	0.075	0.008	0.291
N retention	12.1	14.1	12.9	11.8	11.7	2.96	0.977	0.754	0.716
Utilization of N efficiency (%)	28.8	33.9	34.9	31.8	33.8	7.34	0.978	0.734	0.710

Least square means with different superscript within a row are significantly different (*P* ≤ 0.05)

Digestible OM intake (g/kg DM) = (OM intake - fecal OM)/DM intake

Utilization of N efficiency = (N retention/N intake) × 100

*SEM* standard error of means

## Discussion

The present study compared the nutrient intake and digestibility, rumen fermentation, nitrogen balance and microbial protein synthesis of goats fed increasing dietary levels of HSC in place of SBM. The observed disparities in the experimental diets’ chemical composition were largely ascribed to the substitutive effect of SBM with HSC. A similar trend was reported when HSC was included in lamb finisher diets (Mustafa et al. 1999; Karlsson and Martinsson 2011; Mierliță 2018).

The linear decrease in CP intake and increase in EE intake corresponds to their dietary contents as influenced by the addition of HSC. An earlier study shows that feeding whole hempseed cake to dairy goats did not affect CP intake but increased EE intake (Rapetti et al. 2021). Within the recommended dietary CP and EE range, intake of both proximate components is directly proportional to the dietary contents (Patra 2013; Owens et al. 2014). The increment in EE intake with dietary HSC inclusion despite the tendency of DMI to decline might also be attributed to biohydrogenation of dietary PUFA by *B. fibrisolvens* which enhances fat intake up to 42 g/kg DM dietary fat (Patra 2013; Owens et al. 2014). Of importance, goats have a high number of *B. fibrisolvens* which enables them to tolerate diets with high dietary fat relative to other ruminants (Shivani et al. 2016; Candyrine et al. 2017).

The similarity in NDF intake of goats despite its variation in the nutritional composition of diets suggests that the differences, especially in NDF and tannin contents were not sufficient to influence palatability and digestion. The inclusion of HSC in ruminant diets has been reported to not affect feed palatability (Semwogerere et al. 2020; Addo et al. 2023). More so, the NDF of diets in the current study was less than 180 g/kg DM which is considered to be too low to affect nutrient intake in goats (Avondo et al. 2008). The concentrations of the most dominant polyphenol (i.e., condensed tannins) in HSC were below 55 g CT/kg DM, which is too low to have any adverse effect on ruminants' feed intake and digestibility (Kumar and Singh 1984; Min et al. 2003; Pojić et al. 2014). When HSC is used in place of SBM or canola meal in lambs, similar NDF intake outcomes have also been reported (Mustafa et al. 1999; Antunović et al. 2020).

Similarities in the digestibility values agree with an earlier study with lambs where HSC replaced canola meal (Mustafa et al. 1999). The lack of differences in the digestibility values could be ascribed to the similarities in nutrient intake across diets. The DMI of goats has also previously been reported to be similar when fed 100 g/kg DM of HSC replacing SBM (Abrahamsen et al. 2021; Semwogerere et al. 2022). However, the digestibility values observed in the current study are higher than those reported in lambs fed HSC diets (Mustafa et al. 1999). This species difference may be explained by goats’ superior capacity to digest fibrous diet compared to other ruminants (Domingue et al. 1991; Aregheore 1996).

The similarity in rumen ammonia values across diets observed in the present study despite the linear decline in CP intake could be attributed to the increase in dietary fat intake. Dietary fat deters proliferation of rumen protozoans thus limiting protozoal predation on rumen bacteria thereby reducing bacterial protein recycling (Bach et al. 2005; Patra 2013; Nur Atikah et al. 2018). The current study’s ruminal pH and ammonia are comparable to other goat studies that fed whole hempseed (Rapetti et al. 2021) and SBM-based diets (Gomes et al. 2018). However, the substitution of SBM with HSC up to 30% has been reported to decrease rumen fermentation parameters among goats and this was attributed to the reduction of NFC (Abrahamsen et al. 2021). In the current study, though a linear decline in NFC was recorded among diets, no difference in the rumen fermentation parameters was observed, which might be attributed to lower HSC inclusion levels.

The lack of HSC inclusion effect on total VFA, acetate and propionate could be ascribed to the similar OM intake and digestibility across diets. The increase in OM intake and digestibility has been reported to result in high total VFA and acetate in lambs (Tadayon et al. 2017). Feed organic matter is the main substrate for rumen microbial growth and VFA production (Faverdin 1999; Soltan and Patra 2021). However, Neto et al. (2016) suggested that the acetate concentration is more related to NDF intake and digestibility. In this study, HSC inclusion had no effect on NDF intake and digestibility, which could further explain the similarity in acetate concentration. Butyrate, isobutyrate, valerate and isovalerate are derivatives of branched amino acid fermentation in the rumen (Russell and Sniffen 1984; Van Soest 1994). The amino acid profile of HSC and SBM are comparable (Karlsson et al. 2012; Paula et al. 2018). Therefore, the concentrations of these branched VFA are determined by CP intake and digestibility which was not influenced by addition of HSC to the diets.

The current study is the first to report urinary PD excretions from ruminants fed HSC. The reduction in CP intake might have resulted in low urine volume produced (Van Vuuren and Smits 1997; Dijkstra et al. 2013). The linear reduction in urinary PD excretion and microbial nitrogen supply with increasing levels of HSC could be attributed to a linear decline in NFC, resulting in OM intake tendency to decrease with the addition of HSC to the diet (Ma et al. 2015). The amount of urinary PD excreted is directly proportional to digestible OM in the rumen (DOMR) which is associated with OM intake (Chen and Gomes 1992). Urinary PD excretion is a proxy of microbial protein supply to ruminants (Chen and Gomes 1992; Soliva et al. 2015). A decline in microbial nitrogen with increasing HSC could imply a reduction in DOMR, which subsequently limits microbial growth and their post-ruminal flow, which translates into low urinary PD excretes. More so, most literature relates the decline in urinary PD excretion to anti-quality factors such as condensed tannins as they limit protein degradability and digestibility (Chen and Ørskov 2004; Abarghuei et al. 2010). However, condensed tannin contents of the diets were too low to influence protein digestion in the small intestines as stated earlier. However, the bioavailability of cannabinoids, another group of bioactive compounds in HSC, and their effects on protein digestion in ruminants are not known and merit investigation.

The reduction in urinary N might be attributed to the linear decline in CP intake as urinary N directly correlates with CP intake (Van Vuuren and Smits 1997; Dijkstra et al. 2013). No literature is available on N balance for goats fed HSC. The lack of difference in fecal N, and N retention could be attributed to a similar trend observed for CP digestibility and rumen fermentation parameters. Fecal N and N retention are a function CP digestion, absorption and N recycling (Calsamiglia et al. 2010; Chrenková et al. 2018). These processes are mainly controlled by rumen microbial activity (Nandra et al. 1993; Chrenková et al. 2018). The N efficiency of utilization for diets was within the 10–40 range recommended for highly productive ruminants (Calsamiglia et al. 2010).

Overall, the inclusion of HSC in goat finisher diets linearly decreased their CP intake, microbial N supply, PD absorbed and excreted without affecting rumen fermentation parameters, apparent nutrient digestibility and N retention. It was concluded that HSC could substitute SBM up to 100 g/kg DM in goat finisher diets without compromising nutrient digestibility and utilization. Hempseed cake has proved to be a viable and beneficial feed ingredient in commercial goat finisher diets that could improve production (Semwogerere et al. 2022) and enhance meat health value (Semwogerere et al. 2023a, 2023b) without influencing nutrient digestibility and utilization. Utilization of HSC a livestock feed could enhance the market value of hemp as it provides additional value to the by-product of oil extraction. Further research could be done to verify current findings against different hemp varieties and goat breeds. Additionally, future studies could increase animal numbers and collect rumen fluid on-farm using a stomach tube as these are the main limitations of this study.

## Data Availability

None of the data were deposited in an official repository but data is available upon request from the corresponding author.

## References

[CR1] Abarghuei MJ, Rouzbehan Y, Alipour D (2010). The influence of the grape pomace on the ruminal parameters of sheep. Livestock Science.

[CR2] Abrahamsen FW, Gurung NK, Abebe W, Reddy GP, Mullenix K, Adhikari S (2021). Effects of feeding varying levels of hempseed meal on dry matter intake, rumen fermentation, in vitro digestibility, blood metabolites, and growth performance of growing meat goats. Applied Animal Science.

[CR3] Addo, F., Gervais, R., Ominski, K., Yang, C. and Plaizier, J.C., 2023. Comparing dehulled hemp meal and canola meal as a protein supplement for lactating dairy cows Journal of Dairy Science, TBC No. TBC. 10.3168/jds.2023-23507 (American Dairy Science Association) 10.3168/jds.2023-23507 37641358

[CR4] Aloo SO, Mwiti G, Ngugi LW, Oh DH (2022). Uncovering the secrets of industrial hemp in food and nutrition: The trends, challenges, and new-age perspectives. Critical Reviews in Food Science and Nutrition.

[CR5] Antunović Z, Šalavardić ŽK, Steiner Z, Crossed D, Signidara M, Davar S, Ronta M, Šabić AM, Pavić V, Novoselec J (2020). The influence of hempseed cake on production traits, metabolic profile and antioxidant status of Merinolandschaf lambs Annals of. Animal Science.

[CR6] Horwitz W, AOAC (2002). Official methods of analysis.

[CR7] Aregheore M (1996). Voluntary intake and nutrient digestibility of crop-residue based rations by goats and sheep. Small Ruminant Research.

[CR8] Avondo M, Biondi L, Pagano R, Bonanno A, Lutri L (2008). Feed intake. Dairy goats, feeding and nutrition.

[CR9] Bach A, Calsamiglia S, Stern MD (2005). Nitrogen metabolism in the rumen. Journal of Dairy Science.

[CR10] Brand, T.S., Van Der Merwe, D.A., Raffrenato, E. and Hoffman, L.C., 2020. Predicting the growth and feed intake of Boer goats in a feedlot system South African Journal of Animal Science, 50, 492–500. 10.4314/sajas.v50i4.1

[CR11] Broderick GA, Kang JH (1980). Automated simultaneous determination of ammonia and total amino acids in ruminal fluid and in vitro media. Journal of Dairy Science.

[CR12] Calsamiglia S, Ferret A, Reynolds CK, Kristensen NB, Van Vuuren AM (2010). Strategies for optimizing nitrogen use by ruminants. Animal.

[CR13] Candyrine SCL, Jahromi MF, Ebrahimi M, Liang JB, Goh YM, Abdullah N (2017). In vitro rumen fermentation characteristics of goat and sheep supplemented with polyunsaturated fatty acids Animal Production. Science.

[CR14] Chen XB, Gomes MJ (1992). Estimation of microbial protein supply to sheep and cattle based on urinary excretion of purine derivatives -an overview of the technical details.

[CR15] Chen XB, Ørskov ER, Makkar HPS, Chen XB (2004). Research on Urinary Excretion of Purine Derivatives in Ruminants: Past, Present and Future. Estimation of Microbial Protein Supply in Ruminants Using Urinary Purine Derivatives.

[CR16] Chrenková M, Mlyneková Z, Formelová Z, Polačiková M, Rajský M, Marinov M (2018). Different methods for estimation of protein intestinal digestibility in ruminants. Archiva Zootechnica.

[CR17] Costa ED, Ribiero CV, Silva TM, Ribeiro RD, Vieira JF, Lima AD, Barbosa AM, da Silva Júnior JM, Bezerra LR, Oliveira RL (2021). Intake, nutrient digestibility, nitrogen balance, serum metabolites and growth performance of lambs supplemented with Acacia mearnsii condensed tannin extract. Animal Feed Science and Technology.

[CR18] Dahmer, P.L., McDonald, F.B., Chun, C.K.Y., Zumbaugh, C.A., Jones, C.K., Crane, A.R., Kott, T., Lattimer, J.M. and Chao, M.D., 2022. Evaluating the impact of feeding dried distillers grains with solubles on Boer goat growth performance, meat color stability and antioxidant capacity Translational Animal Science, 6, 1–9. 10.1093/tas/txac060 10.1093/tas/txac060 PMC9186308 35702176

[CR19] Dijkstra, J., Oenema, O., van Groenigen, J.W., Spek, J.W., van Vuuren, A.M. and Bannink, A., 2013. Diet effects on urine composition of cattle and N2O emissions. Animal : an international journal of animal bioscience, 7, 292–302. 10.1017/S1751731113000578 (Elsevier) 10.1017/S1751731113000578 23739471

[CR20] Domingue FBM, Dellow DW, Barry TN (1991). The efficiency of chewing during eating and ruminating in goats and sheep. British Journal of Nutrition.

[CR21] Embaby, M.G., Günal, M. and Abughazaleh, A., 2019. Effect of unconventional oils on in vitro rumen methane production and fermentation International Journal of Agricultural and Natural Resources, 46, 276–285. 10.7764/rcia.v46i3.2062

[CR22] FAOSTAT, 2022. Global hempseed production, https://www.fao.org/faostat/en/#data/QCL Accessed on 05 November 2022. (Rome, Italy)

[CR23] Faverdin P (1999). The effect of nutrients on feed intake in ruminants. Proceedings of the Nutrition Society.

[CR24] Frassinetti S, Moccia E, Caltavuturo L, Gabriele M, Longo V, Bellani L, Giorgi G, Giorgetti L (2018). Nutraceutical potential of hemp (Cannabis sativa L.) seeds and sprouts. Food Chemistry.

[CR25] Freer M, Dove H, Nolan JV, Freer M, Dove H, Nolan JV (2007). Nutrient requirements of domesticated ruminants.

[CR26] Gomes LC, Alcalde CR, Damasceno JC, Rigolon LP, Possamai APS, Hygino B (2018). Concentrate containing calcium salts of fatty acids rich in polyunsaturated fatty acids can change rumen fermentation in grazing goats. Semina:Ciencias Agrarias.

[CR27] Hall MB (2009). Analysis of starch, including maltooligosacchardies in animal feeds: A comparison of methods and a recommended method for AOAC collaborative study. Journal of AOAC International.

[CR28] Harper K, McNeill D (2015). The role iNDF in the regulation of feed intake and the importance of its assessment in subtropical ruminant systems (the role of iNDF in the regulation of forage intake). Agriculture.

[CR29] Horne MRL, Kozłowski RM, Mackiewicz-Talarczyk M (2020). Bast fibres: hemp cultivation and production. Handbook of Natural Fibres (Second Edition).

[CR30] Karlsson L, Martinsson K (2011). Growth performance of lambs fed different protein supplements in barley-based diets. Livestock Science.

[CR31] Karlsson L, Ruiz-Moreno M, Stern MD, Martinsson K (2012). Effects of temperature during moist heat treatment on ruminal degradability and intestinal digestibility of protein and amino acids in hempseed cake. Asian-Australasian Journal of Animal Sciences.

[CR32] Kumar R, Singh M (1984). Tannins: Their adverse role in ruminant nutrition. Journal of Agricultural and Food Chemistry.

[CR33] Kustantinah, K., Suhartanto, B., Indarto, E., Zulfa, I.H. and Atmojo, F.A., 2020. Degradation of nitrogen fraction in kacang goats feed supplementation Calliandra calothyrsus substituted soybean meal Key Engineering Materials, 840, 118–123. 10.4028/www.scientific.net/kem.840.118

[CR34] Leonard W, Zhang P, Ying D, Fang Z (2020). Hempseed in food industry: Nutritional value, health benefits, and industrial applications. Comprehensive Reviews in Food Science and Food Safety.

[CR35] Lu CD, Kawas JR, Mahgoub OG (2008). Recent advancements in fiber digestion and utilization in goats. Tropical and subtropical agroecosystems.

[CR36] Ma T, Tu Y, Zhang NF, Deng KD, Diao QY (2015). Effect of the ratio of non-fibrous carbohydrates to neutral detergent fiber and protein structure on intake, digestibility, rumen fermentation, and nitrogen metabolism in lambs. Asian-Australasian Journal of Animal Sciences.

[CR37] Makkar HPS (2003). Quantification of tannins in tree and shrub foliage. A laboratory manual.

[CR38] Mierliță D (2018). Effects of diets containing hemp seeds or hemp cake on fatty acid composition and oxidative stability of sheep milk South African. Journal of Animal Science.

[CR39] Min BR, Barry TN, Attwood GT, McNabb WC (2003). The effect of condensed tannins on the nutrition and health of ruminants fed fresh temperate forages: A review. Animal Feed Science and Technology.

[CR40] Moscariello C, Matassa S, Esposito G, Papirio S (2021). From residue to resource: The multifaceted environmental and bioeconomy potential of industrial hemp (Cannabis sativa L.). Resources, Conservation and Recycling.

[CR41] Mustafa AF, McKinnon JJ, Christensen DA (1999). The nutritive value of hemp meal for ruminants. Canadian Journal of Animal Science.

[CR42] Nandra KS, Hendry A, Dobos RC (1993). A study of voluntary intake and digestibility of roughages in relation to their degradation characteristics and retention time in the rumen. Animal Feed Science and Technology.

[CR43] National Research Council (2007). Nutrient Requirements of Small Ruminants: Sheep, Goats, Cervids, and New World Camelids, National Research Council (U.S.). Committee on Nutrient Requirements of Small Ruminants (ed).

[CR44] Neto, G.O.J.D.A., Parente, M.D.O.M., Parente, H.N., Alves, A.A., Santos, P.A.C. Dos, Moreira Filho, M.A., Zanine, A.D.M., Ferreira, D.D.J., Bezerra, L.R. and Gomes, R.M.D.S., 2016. Intake, nutrient apparent digestibility, and ruminal constituents of crossbred Dorper × Santa Inês sheep fed diets with babassu mesocarp flour Scientific World Journal, 2016, 1-8. 10.1155/2016/8675836 10.1155/2016/8675836 PMC5124479 27957525

[CR45] Nur Atikah I, Alimon AR, Yaakub H, Abdullah N, Jahromi MF, Ivan M, Samsudin AA (2018). Profiling of rumen fermentation, microbial population and digestibility in goats fed with dietary oils containing different fatty acids. BMC Veterinary Research.

[CR46] Owens FN, Qi S, Sapienza DA (2014). INVITED REVIEW: Applied protein nutrition of ruminants-Current status and future directions Professional Animal. Scientist.

[CR47] Pantoja J, Firkins JL, Eastridge ML, Hull BL (1994). Effects of fat saturation and source of fiber on site of nutrient digestion and milk production by lactating dairy cows. Journal of Dairy Science.

[CR48] Patra AK (2013). The effect of dietary fats on methane emissions, and its other effects on digestibility, rumen fermentation and lactation performance in cattle: A meta-analysis. Livestock Science.

[CR49] Paula EM, Broderick GA, Danes MAC, Lobos NE, Zanton GI, Faciola AP (2018). Effects of replacing soybean meal with canola meal or treated canola meal on ruminal digestion, omasal nutrient flow, and performance in lactating dairy cows. Journal of Dairy Science.

[CR50] Pojić M, Mišan A, Sakač M, Hadnađev DT, Šarić B, Milovanović I, Hadnađev M (2014). Characterization of byproducts originating from hemp oil processing. Journal of Agricultural and Food Chemistry.

[CR51] Rapetti, L., Colombini, S., Battelli, G., Castiglioni, B., Turri, F., Galassi, G., Battelli, M. and Crovetto, G.M., 2021. Effect of linseeds and hemp seeds on milk production, energy and nitrogen balance, and methane emissions in the dairy goat Animals, 2021, 1-19. 10.3390/ani11092717 10.3390/ani11092717 PMC8470940 34573683

[CR52] Ricci C, Baumgartner J, Malan L, Smuts CM (2020). Determining sample size adequacy for animal model studies in nutrition research: limits and ethical challenges of ordinary power calculation procedures. International Journal of Food Sciences and Nutrition.

[CR53] Russell JB, Sniffen CJ (1984). Effect of Carbon-4 and Carbon-5 volatile fatty acids on growth of mixed rumen bacteria In vitro. Journal of Dairy Science.

[CR54] Ryan MG, Melillo JM, Ricca A (1990). A comparison of methods for determining proximate carbon fractions of forest litter. Canadian Journal of Forest Research.

[CR55] Sainz Martinez A, Lanaridi O, Stagel K, Halbwirth H, Schnürch M, Bica-Schröder K (2023). Extraction techniques for bioactive compounds of cannabis. Natural Product Reports.

[CR56] Santos SA, Prates LL, de Carvalho GG, dos Santos AC, de Campos Valadares Filho S, Tosto MS, Mariz LD, da Silva Neri F, de Queiroz Sampaio M (2017). Creatinine as a metabolic marker to estimate urinary volume in growing goats. Small Ruminant Research.

[CR57] Semwogerere F, Chikwanha OC, Katiyatiya CL, Marufu MC, Mapiye C (2023). Bioavailability of bioactive phytochemicals in selected tissues and excreta from goats fed hempseed cake (Cannabis sativa L.) finisher diets. Tropical animal health and production.

[CR58] Semwogerere F, Chikwanha OC, Katiyatiya CLF, Marufu MC, Mapiye C (2022). Chevon production and quality of Kalahari Red goats fed increasing levels of hempseed cake substituted for soybean meal. Meat Science.

[CR59] Semwogerere F, Chikwanha OC, Katiyatiya CLF, Marufu MC, Mapiye C (2023). Health value and keeping quality of chevon from goats fed finisher diets containing hemp (Cannabis sativa L.) seed cake. Meat Science.

[CR60] Semwogerere F, Katiyatiya CLF, Chikwanha OC, Marufu MC, Mapiye C (2020). Bioavailability and Bioefficacy of Hemp By-Products in Ruminant Meat Production and Preservation: A Review Frontiers in Veterinary. Science.

[CR61] Shapiro SS, Wilk MB (1965). An Analysis of Variance Test for Normality ( Complete Samples ). Biometrika.

[CR62] Shivani S, Srivastava A, Shandilya UK, Kale V, Tyagi AK (2016). Dietary supplementation of Butyrivibrio fibrisolvens alters fatty acids of milk and rumen fluid in lactating goats. Journal of the Science of Food and Agriculture.

[CR63] Siano F, Moccia S, Picariello G, Russo GL, Sorrentino G, Di Stasio M, La Cara F, Volpe MG (2019). Comparative study of chemical, biochemical characteristic and ATR-FTIR analysis of seeds, oil and flour of the edible Fedora cultivar hemp (Cannabis sativa L.). Molecules.

[CR64] Siegfried VR, Ruckermann H, Stumpf G, Siegfried BD, Ruckemann H, Siegfried R, Siegfried MR (1984). Method for the determination of organic acids in silage by high performance liquid chromatography. Landwirtsch Forsch.

[CR65] Soliva CR, Amelchanka SL, Kreuzer M (2015). The requirements for rumen-degradable protein per unit of fermentable organic matter differ between fibrous feed sources. Frontiers in Microbiology.

[CR66] Soltan YA, Patra AK, Patra AK (2021). Ruminal microbiome manipulation to improve fermentation efficiency in ruminants. Animal Feed Science and Nutrition - Production, Health and Environment [Working Title].

[CR67] South African government Gazzette (2000). Meat safety act, (Parliament of the republic of South Africa: Pretoria South Africa, South Africa).

[CR68] Tadayon Z, Rouzbehan Y, Rezaei J (2017). Effects of feeding different levels of dried orange pulp and recycled poultry bedding on the performance of fattening lambs1. Journal of Animal Science.

[CR69] Udén P, Robinson PH, Mateos GG, Blank R (2012). Use of replicates in statistical analyses in papers submitted for publication in Animal Feed Science and Technology. Animal Feed Science and Technology.

[CR70] Van Soest PJ, Van Soest PJ (1994). Nutritional Ecology of the Ruminant.

[CR71] Van Vuuren AM, Smits MCJ, Jarvis S, Pain B (1997). Effect of nitrogen and sodium chloride intake on production and composition of urine in dairy cows. Gaseous nitrogen emissions from grasslands.

[CR72] Wang S, Kreuzer M, Braun U, Schwarm A (2017). Effect of unconventional oilseeds (safflower, poppy, hemp, camelina) on in vitro ruminal methane production and fermentation. Journal of the Science of Food and Agriculture.

[CR73] Winders TM, Holman DB, Schmidt KN, Luecke SM, Smith DJ, Neville BW, Dahlen CR, Swanson KC, Amat S (2023). Feeding hempseed cake alters the bovine gut, respiratory and reproductive microbiota. Scientific Reports.

[CR74] Wiseman J (2018). Editorial: Digestibility and degradability in animal nutrition studies. Journal of Agricultural Science.

